# Epstein–Barr virus and cytomegalovirus reactivation after allogeneic hematopoietic cell transplantation in patients with non–Hodgkin lymphoma: the prevalence and impacts on outcomes: EBV and CMV reactivation post allo-HCT in NHL

**DOI:** 10.1007/s00277-021-04642-5

**Published:** 2021-09-04

**Authors:** Yiyang Ding, Yuhua Ru, Tiemei Song, Lingchuan Guo, Xiang Zhang, Jinjin Zhu, Caixia Li, Zhengming Jin, Haiwen Huang, Yuqing Tu, Mimi Xu, Yang Xu, Jia Chen, Depei Wu

**Affiliations:** 1 National Clinical Research Center for Hematologic Diseases, Jiangsu Institute of Hematology, The First Affiliated Hospital of Soochow University, Shizi Street 188, Suzhou, 215006 China; 2 Institute of Blood and Marrow Transplantation, Collaborative Innovation Center of Hematology, Soochow University, Suzhou, China; 3 Key Laboratory of Stem Cells and Biomedical, Materials of Jiangsu Province and Chinese Ministry of Science and Technology, Suzhou, China; 4 The pathology department of the First Affiliated Hospital of Soochow University, Suzhou, China

**Keywords:** Epstein–Barr virus, Cytomegalovirus, Non-Hodgkin lymphoma, Allogeneic hematopoietic cell transplantation

## Abstract

Epstein–Barr virus (EBV) and cytomegalovirus (CMV) reactivations are common complications after allogeneic hematopoietic cell transplantation (allo-HCT), but data focusing on non–Hodgkin lymphoma (NHL) are limited. We retrospectively analyzed the prevalence of EBV and CMV reactivation post-allo-HCT and the impacts on transplant outcomes in 160 NHL patients. The 1-year incidences of EBV and CMV reactivation were 22.58% and 25.55%, respectively. Independent impactors for EBV reactivation were more than 6 lines of chemotherapy (*P* = 0.030), use of rituximab (*P* = 0.004), and neutrophil recovery within 30 days post-HCT (*P* = 0.022). For T-cell lymphoblastic lymphoma patients, the International Prognostic Index (IPI) (*P* = 0.015) and chronic GVHD (*P* = 0.001) increased the risk of CMV reactivation. CMV reactivation was independently related to a lower risk of relapse (*P* = 0.027) but higher transplant-related mortality (TRM) (*P* = 0.038). Although viral reactivation had no significant impact on overall survival (OS) in the whole cohort, it led to an inferior 2-year OS (67.6% versus 92.5%, *P* = 0.005) and TRM (20.1% versus 4.7%, *P* = 0.020) in recipients surviving for more than 180 days. We concluded that EBV and CMV reactivation post-allotransplant still deserved concern particularly in NHL patients with high-risk factors, since it is generally related to a deteriorated prognosis. Large-scale studies are warranted to validate our findings.

**Supplementary Information:**

The online version contains supplementary material available at 10.1007/s00277-021-04642-5.

## Introduction

Patients with relapsed and refractory (R/R) non–Hodgkin lymphoma (NHL) have a dismal prognosis. Despite emerging agents and cellular therapies, allogeneic hematopoietic cell transplantation (allo-HCT) remains an essential modality to attain long-term survival [1–3]. However, transplant outcomes are impaired by all kinds of complications.

Epstein–Barr virus (EBV) and cytomegalovirus (CMV) reactivations are frequent complications after allo-HCT that could cause fatal virus-related diseases [4–6]. Moreover, EBV per se has been causally linked to the pathogenesis of several types of NHL [7, 8] or posttransplant lymphoproliferative diseases (PTLDs). The reported incidences fluctuate widely from 0.1 to 63% for EBV [9] and from 30 to 70% for CMV reactivation after HCT [10–13] with ambiguous impacts on transplant outcomes [14–18], but limited data focusing on NHL patients have been reported. Hence, we conducted a retrospective analysis to investigate the features of EBV and CMV reactivation after allo-HCT in NHL patients.

## Materials and methods

### Patients

This was a retrospective study based on data from the transplant database in our center, which was established according to the European Society for Blood and Marrow Transplantation registry. The inclusion criteria included (1) patients who were histologically diagnosed with NHL; (2) patients who underwent allo-HCT between January 2010 and December 2018; and (3) patients who received regular EBV and CMV monitoring after HCT based on an institutional protocol. The study protocol was approved by the Ethics Committee of our center and conducted in accordance with the Helsinki Declaration.

### Transplant protocol

NHL patients with the following indications were recommended to receive allo-HCT in our center: (1) refractory to more than 2 lines of chemotherapy; (2) relapsed within 1 year after the completion of treatment, or had a history of autologous HCT; (3) lymphoblastic lymphoma (LBL), highly aggressive T-cell NHL, or transformed diffuse large B-cell lymphoma (DLBCL) arising from follicular lymphoma or chronic lymphocytic leukemia, etc. Donor selection was based on HLA typing, age, donor sex, ABO compatibility, and physical health [19]. An HLA-matched sibling was preferred, and a matched unrelated donor, a haploidentical donor, or umbilical cord blood units could be an alternative option [20]. Donors were encouraged to contribute a bone marrow graft, and peripheral blood stem cells were collected if the CD34 + cell dose was less than the target dose of 2 × 10^6^/kg of recipient body weight. All patients in this cohort received myeloablative conditioning (MAC), including the modified Bu/Cy regimen and the modified TBI/Cy regimen [21].

The prophylaxis of graft-versus-host disease (GVHD) was included cyclosporin A (CsA) and short-term methotrexate for recipients receiving HLA-matched sibling donor grafts, and mycophenolate mofetil (MMF) combined with antithymocyte globulin (ATG) (Genzyme, MA, USA) [22] was added to unrelated or haploidentical donor HCT. Acute and chronic GVHD was diagnosed according to reference literature [23, 24].

### Management of virus reactivation

Q-PCR was applied to monitor EBV-DNA and CMV-DNA load in whole peripheral blood weekly from conditioning to + 90 days post-HCT in all patients and once every 2 weeks from + 90 days until + 180 days. Additional detection was performed if symptoms of suspected virus infection were present in individual situations. Ganciclovir 5 mg/kg twice a day or foscarnet 90 mg/kg twice a day was routinely used from − 9 to − 2 days to prevent virus infection and then replaced by acyclovir to avoid marrow toxicity. The treatment for reactivation included ganciclovir, foscarnet, and tapering of immunosuppressive agents. Preemptive rituximab was prescribed if EBV-DNA reached 10^5^ copies/mL or 10^4^ copies/mL for 2 consecutive weeks.

### Definition

EBV and CMV reactivation was defined as more than 10^2^ copies/mL DNA load in our center. Neutrophil recovery was defined as the first day when neutrophil count was above 0.5 × 10^9^/L for three consecutive days after HCT, and platelet recovery was defined as the first day when the platelet count was above 20 × 10^9^/L for seven consecutive days without transfusion. Advanced disease status at transplant was defined as all disease statuses except complete remission (CR). OS was defined as the duration from transplantation to death from any cause. Progression-free survival (PFS) was defined as survival without disease relapse or progression. Deaths unrelated to the underlying disease were recorded as transplant-related mortality (TRM). GRFS was defined as survival in the absence of grade II–IV acute GVHD, extensive chronic GVHD, relapse, or death from any cause after allo-HCT.

### Statistics

The incidence of virus reactivation, OS, PFS, and graft-versus-host disease-free and relapse-free survival (GRFS) was calculated using the Kaplan–Meier method and compared with the log-rank test. The cumulative incidence of disease relapse or progression (CIR) was calculated by a competing risk model with TRM as a competing risk factor. Risk analyses were conducted by the Cox regression model, and all risk factors whose *P* values were below 0.1 in univariate analyses were included in multivariate analyses. EBV and CMV reactivations were treated as time-dependent variables in the risk analyses. Since neutrophil recovery was correlated with platelet recovery (Pearson correlation coefficient of 0.40, *P* < 0.001), only neutrophil recovery was enrolled in multivariate analysis if *P* value of both variables was below 0.1 in univariate analyses. All tests were two-sided, and *P* values < 0.05 were considered statistically significant. Statistical analyses were performed using SPSS 22.0 software (SPSS, Chicago, IL, USA) and the R 3.6.2 software package (The R Foundation for Statistical Computing, Vienna, Austria).

## Results

### Patient characteristics

A total of 160 patients were included according to the inclusion criteria, and the median time from diagnosis to transplantation was 8 months. The patient characteristics are summarized in Table 1. The enrolled patients consisted of 107 males and 53 females, with a median age of 30 (range, 5–59) years old at the time of allo-HCT. Of the 160 cases, 85 were LBL, 23 were DLBCL, 13 were peripheral T cell lymphoma, 10 were NK/T cell lymphoma, 7 were Burkitt lymphoma, 6 were anaplastic large cell lymphoma, 4 were mantle cell lymphoma, 3 were aggressive NK cell lymphoma, 2 were Richter syndrome, 2 were high-grade B-cell lymphoma, 2 were liver and spleen γδT-cell lymphoma, 1 was angioimmunoblastic lymphoma, 1 was follicular lymphoma (grade 3), and 1 was gray zone lymphoma. Nine patients received an autologous HCT before allo-HCT with a median interval of 11 months between the two transplants, and 7 patients received a previous CAR-T cell therapy. Only 43 patients had matched-related donors, while others received grafts from HLA-matched unrelated donors (*n* = 23), haploidentical related donors (*n* = 90), HLA-mismatched donors (*n* = 2), and HLA-mismatched umbilical cord blood (*n* = 2).

**Table 1:** Characteristics of patients undergoing allogeneic HCT

	Cases	EBV +	CMV +
Sex			
Male	107	24	28
Female	53	11	12
Median age (year)			
30			
Lymphoma classification			
B-cell lymphoblastic lymphoma	22	6	7
Non-lymphoblastic B-cell lymphoma	40	1	12
T-cell lymphoblastic lymphoma	63	14	13
Non-lymphoblastic T-cell lymphoma	35	14	8
Autologous HCT before allo-HCT			
No	151	31	36
Yes	9	4	4
CAR-T cell therapy before allo-HCT			
No	153	34	39
Yes	7	1	1
Disease status before allo-HCT			
CR	89	15	20
Advanced status	71	20	20
Donor type			
HLA-matched donors	66	8	12
HLA-mismatched donors	94	27	28
Type of graft			
BM	14	3	3
PB	65	14	12
BM + PB	79	18	24
dUCB	2	0	1
IPI stratification			
Low risk	32	5	5
Low-intermediate risk	85	21	21
High-intermediate risk	38	7	13
High risk	5	2	1
NCCN-IPI stratification			
Low risk	25	3	5
Low-intermediate risk	109	29	29
High-intermediate risk	26	3	6
Ann Arbor			
I	5	1	1
II	6	2	0
III	14	3	4
IV	135	29	35
Time from diagnosis to HCT			
< 8 m	79	16	15
≥ 8 m	81	19	25
Chemotherapy lines			
< 6	78	11	14
≥ 6	82	24	26
ATG use			
No	54	4	10
Yes	106	31	30
TBI use			
No	108	24	15
Yes	52	11	25
Rituximab			
No	117	34	27
Yes	43	1	13
Prophylactic therapy			
Ganciclovir	83	20	21
Foscarnet	50	11	12
Acyclovir	27	4	7
Neutrophil recovery within 30 days			
No	7	2	2
Yes	153	33	38
Platelet recovery within 60 days			
No	37	9	31
Yes	123	26	9
Acute GVHD			
None	82	19	17
Acute GVHD	78	16	23
None, grade I	96	22	19
Grades II–IV	64	13	21
Chronic GVHD			
None	115	28	27
Chronic GVHD	45	7	13
None, limited	139	30	33
Extensive	21	5	7

Abbreviations: *NHL* non–Hodgkin lymphoma; *EBV* Epstein–Barr virus; *CMV* human cytomegalovirus; *CR* complete remission; *BM* bone marrow; *PB* peripheral blood; *dUCB* double umbilical cord blood graft; *IPI* the International Prognostic Index; *ATG* antithymocyte globulin; *TBI* total body irradiation; *GVHD* graft-versus-host disease

### Prevalence of virus reactivation

EBV-DNA and CMV-DNA detection was performed in all the donors and recipients before HCT, and the results were negative except for 4 recipients who were EBV-positive. Two of the 4 recipients received rituximab and became negative before transplant, and the other two remained positive even after HCT.

**Fig. 1:**
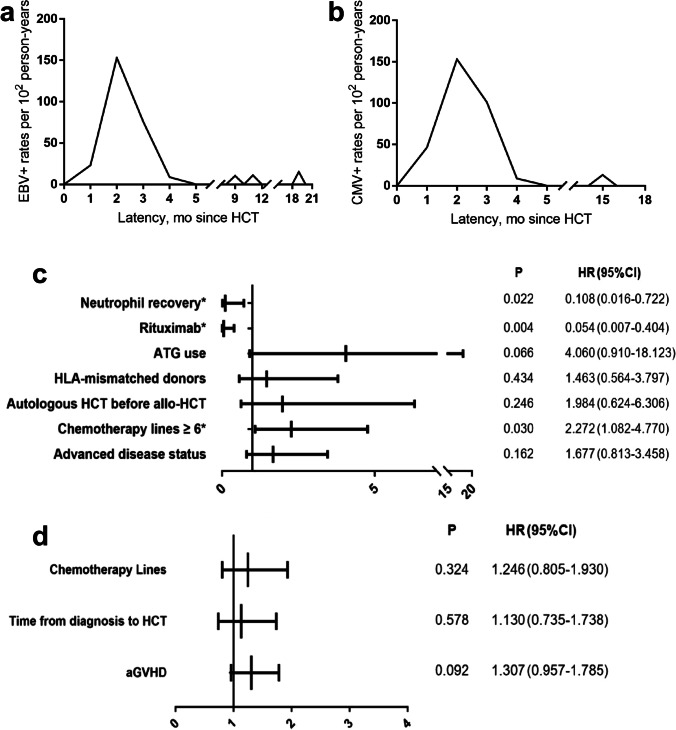
Incidences and risk factors of viral reactivation. **a** Variation tendency of EBV reactivation incidence rate over time. **b** Variation tendency of CMV reactivation incidence rate over time. **c** Multivariate Analysis of EBV reactivation. **d** Multivariate analysis of CMV reactivation. *Significant differences are marked with an asterisk at the *P* value stated

Thirty-five recipients developed EBV reactivation after HCT, while 40 developed CMV reactivation, with a median time of 55 (IQR 43–69) days and 51 (IQR 36.5–62.5) days after HCT, respectively. Viral reactivation after 100 days post-HCT occurred in only 6 patients (3 with EBV reactivation and another 3 with CMV reactivation). The 1-year incidences of EBV and CMV reactivation were similar as 22.58% ± 3.48% and 25.55% ± 3.59%, respectively (Fig. 1). Co-reactivation of EBV and CMV was observed in 10 patients. The 1-year incidence of EBV reactivation in patients with B-cell NHL was significantly lower than that in patients with T-cell NHL (12.28% ± 4.38% vs 28.96% ± 4.82%, *P* = 0.025).

In the subgroup analysis of B-cell NHL, the 100-day incidence of EBV reactivation was markedly decreased in the non-LBL group compared to the LBL group (2.78% ± 2.74% vs 28.57% ± 9.86%, *P* = 0.023) (Table 2). In contrast, the incidence of EBV reactivation was lower in T-cell LBL than that in non-LBL T-cell lymphoma (21.48% ± 5.29% vs 36.93% ± 8.87%, *P* = 0.038). In patients receiving rituximab pre-HCT (*n* = 43), only one patient experienced EBV reactivation on day + 49 post-HCT.

**Table 2:** The 100-day incidence of virus reactivation post-HCT in subgroup analysis

	Incidence		*P*
	T-cell	B-cell	
EBV			
lymphoblastic lymphoma	21.48% ± 5.29%	28.57% ± 9.86%	0.516
Non-lymphoblastic lymphoma	36.93% ± 8.87%	2.78% ± 2.74%	0.004
*P*	0.038	0.023	
CMV			
Lymphoblastic lymphoma	18.04% ± 4.93%	31.82% ± 9.93%	0.256
Non-lymphoblastic lymphoma	23.40% ± 7.78%	30.02% ± 7.58%	0.687
***P***	0.550	0.964	

The incidences of CMV reactivation were similar among different subgroups (Table 2). The 1-year incidences of CMV reactivation among patients who used ganciclovir (*n* = 83), foscarnet (*n* = 50), or acyclovir alone (*n* = 27) as prophylaxis were comparable (27.26% ± 5.10% vs 25.97% ± 6.46% vs 25.17% ± 9.12%, *P* = 0.998).

### Risk factors for virus reactivation

In the univariate analysis, more than 6 lines of chemotherapy (*P* = 0.023), advanced disease status pre-HCT (P = 0.031), HLA-mismatched donors (*P* = 0.021), and the use of ATG (P = 0.006) were associated with EBV reactivation after HCT, while the use of rituximab (*P* = 0.010) was a protective factor (Online Resource1). Neutrophil recovery within 30 days post-HCT (*P* = 0.095) and autologous HCT before allo-HCT (*P* = 0.051) had marginal significance and were included in multivariate analysis. The multivariate analysis identified that more than 6 lines of chemotherapy (HR = 2.272, 95% CI: 1.082–4.770, *P* = 0.030) independently increased the risk of EBV reactivation, while the use of rituximab (HR = 0.054, 95% CI: 0.007–0.404, *P* = 0.004) and neutrophil recovery within 30 days (HR = 0.108, 95% CI: 0.016–0.722, *P* = 0.022) were independent protective factors (Fig. 1). Although no statistically significant risk factors were found for CMV reactivation in the whole cohort (Online Resource1, Fig. 1), the International Prognostic Index (IPI) (*P* = 0.015) and chronic GVHD (*P* = 0.001) were independent risk factors in T-cell LBL patients (Online Resource2).

### GVHD, relapse, and TRM

Acute GVHD occurred in 89 recipients, of whom 64 (71.9%) were grades II–IV and 40 (44.9%) were grades III–IV. Chronic GVHD occurred in 45 patients, 21 of whom were extensive, with a median onset time of 183 (range, 110–1762) days after HCT. EBV reactivation was not associated with the occurrence of GVHD, but CMV reactivation was related to higher grade III–IV acute GVHD (HR = 2.666, 95% CI: 1.153–6.614, *P* = 0.022).

**Fig. 2:**
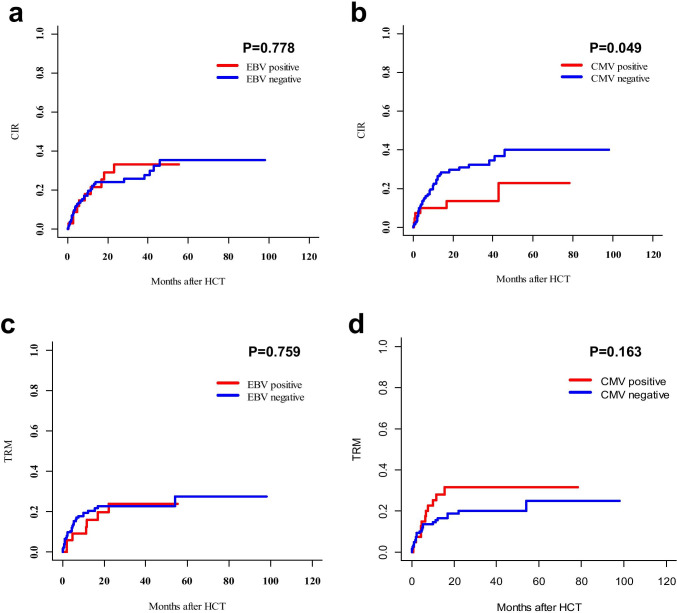
Comparison of CIR and TRM for patients with or without virus reactivation after allo-HCT. **a** CIR of patients with or without EBV reactivation. **b** CIR of patients with or without CMV reactivation. **c** TRM of patients with or without EBV reactivation. **d** TRM of patients with or without CMV reactivation

With a median follow-up for survivors of 21 months, the 2-year CIR of CMV-positive patients was decreased compared to that of CMV-negative patients (13.7 ± 0.3% versus 30.8 ± 0.2%, *P* = 0.049) (Fig. 2b), and the 2-year TRM was comparable (31.4 ± 0.1% versus 19.9 ± 0.0%, *P* = 0.163) (Fig. 2d). Meanwhile, there were no significant differences in CIR (*P* = 0.778) and TRM (*P* = 0.759) between EBV-positive and EBV-negative patients (Fig. 2a, c). Only one patient developed and died from PTLD.

In univariate analysis, CMV reactivation (*P* = 0.026) and neutrophil recovery within 30 days (*P* = 0.039) were associated with an improved CIR. However, only CMV reactivation ameliorated the CIR (HR = 0.265, 95% CI: 0.081–0.860, *P* = 0.027) in multivariate analysis (Table 3). In addition, CMV reactivation (*P* = 0.040), ≥ 8 months from diagnosis to HCT (*P* = 0.042), and advanced disease status (*P* = 0.011) were significant risk factors for TRM in univariate analysis, while chronic GVHD (*P* = 0.030), neutrophil recovery within 30 days (*P* = 0.022), and platelet recovery within 60 days (*P* < 0.001) were related to an improved TRM (Online Resource3). The results of multivariate analysis showed that only CMV reactivation (HR = 2.257, 95% CI: 1.046–4.869, *P* = 0.038) had a remarkable hazardous influence on TRM, while neutrophil recovery within 30 days was identified as an independent protective factor (HR = 0.189, 95% CI: 0.049–0.723, *P* = 0.015).

**Table 3:** Multivariate Cox regression models about association between variables and outcomes

	HR	95%CI		*P*
		Lower limit	Upper limit	
OS				
EBV: positive vs negative	1.479	0.738	2.963	0.270
CMV: positive vs negative	1.789	0.922	3.472	0.086
Disease status: advanced status vs CR	1.800	0.997	3.249	0.051
TBI use: yes vs no	1.419	0.800	2.518	0.232
Neutrophil recovery within 30 days: yes vs no	0.190	0.073	0.499	0.001
Chronic GVHD: chronic GVHD vs none	0.303	0.125	0.733	0.008
PFS				
Neutrophil recovery within 30 days: yes vs no	0.192	0.082	0.447	< 0.001
Disease status: advanced status vs CR	1.643	1.055	2.559	0.028
CIR				
CMV: positive vs negative	0.265	0.081	0.860	0.027
NCCN-IPI stratification				0.120
Low risk	1			
Low-intermediate risk	1.529	0.640	3.652	0.339
High-intermediate risk	0.480	0.120	1.922	0.300
Neutrophil recovery within 30 days: yes vs no	0.359	0.109	1.181	0.092
TRM				
CMV: positive vs negative	2.257	1.046	4.869	0.038
Disease status: advanced status vs CR	1.520	0.714	3.233	0.277
Time from diagnosis to HCT: ≥ 8 m vs < 8 m	1.823	0.830	4.005	0.135
Rituximab: yes vs no	1.847	0.912	3.740	0.088
Neutrophil recovery within 30 days: yes vs no	0.189	0.049	0.723	0.015
Chronic GVHD: chronic GVHD vs none	0.428	0.159	1.149	0.092
GRFS				
EBV: positive vs negative	1.575	0.932	2.661	0.089
CMV: positive vs negative	1.741	1.035	2.927	0.037
IPI stratification				0.024
Low risk	1			
Low-intermediate risk	0.218	0.080	0.594	0.003
High-intermediate risk	0.347	0.138	0.875	0.025
High risk	0.300	0.114	0.791	0.015
Neutrophil recovery within 30 days: yes vs no	0.325	0.148	0.712	0.005

Abbreviations: *OS* overall survival; *PFS* progression-free survival; *CIR* cumulative incidence of relapse; *TRM* treatment-related mortality; *GRFS* graft-versus-host disease-free with relapse-free survival

### OS, PFS, and GRFS

**Fig. 3:**
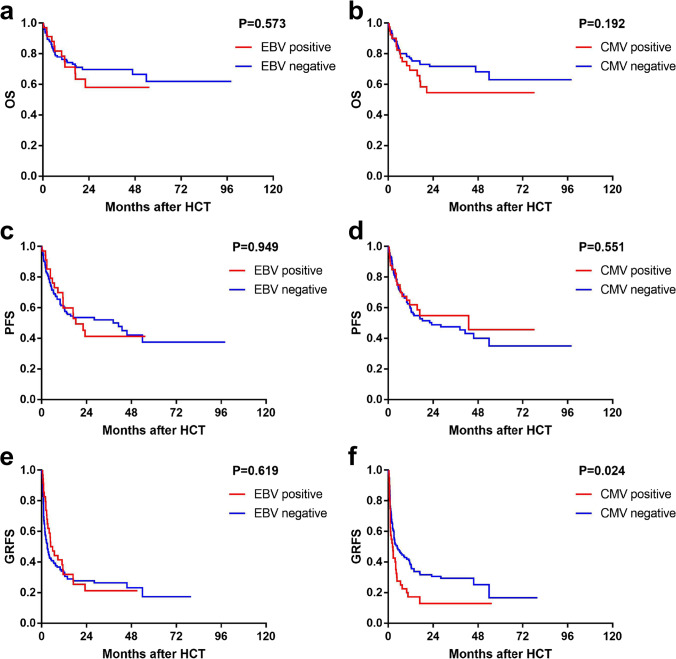
Comparison of OS, PFS and GRFS for patients with or without virus reactivation after allo-HCT. **a** OS of patients with or without EBV reactivation. **b** OS of patients with or without CMV reactivation. **c** PFS of patients with or without EBV reactivation. **d** PFS of patients with or without CMV reactivation. **e** GRFS of patients with or without EBV reactivation. **f** GRFS of patients with or without CMV reactivation

Neither EBV nor CMV reactivation had a significant impact on OS (2-year OS: 58.1% ± 9.7% for EBV-positive patients versus 69.2% ± 4.3% for EBV negative patients, *P* = 0.573; 54.6% ± 8.8% for CMV-positive patients versus 71.7% ± 4.4% for CMV negative patients, *P* = 0.192, respectively) or PFS (2-year PFS: 41.1% ± 9.2% for EBV-positive patients versus 53.1% ± 4.6% for EBV-positive patients, *P* = 0.949; 54.9% ± 8.4% CMV-positive patients versus 48.6% ± 4.9% for CMV negative patients, *P* = 0.551, respectively) (Fig. 3). Chronic GVHD (HR = 0.303, 95% CI: 0.125–0.733, *P* = 0.008) and neutrophil recovery within 30 days (HR = 0.190, 95% CI: 0.073–0.499, *P* = 0.001) were associated with superior OS in multivariate analysis. Advanced disease status (HR = 0.192, 95% CI: 0.082–0.447, *P* < 0.001) and neutrophil recovery within 30 days (HR = 1.643, 95% CI: 1.055–2.559, *P* = 0.028) were independent factors related to PFS in multivariate analysis (Table 3).

The CMV-positive group exhibited an inferior GRFS compared to the CMV-negative group (2-year GRFS: 12.9% ± 5.9% versus 30.3% ± 4.3%, *P* = 0.024) (Fig. 3f), while no marked difference in GRFS was observed between the EBV-positive and EBV-negative groups (2-year GRFS: 21.3% ± 7.5% versus 27.5% ± 4.1%, *P* = 0.619) (Fig. 3e). CMV reactivation (HR = 1.741, 95% CI: 1.035 – 2.927, *P* = 0.037), IPI (*P* = 0.024), and neutrophil recovery at 30 days (HR = 0.325, 95% CI: 0.148–0.712, *P* = 0.005) were independent predictors of GRFS in multivariate analysis (Table 3).

### Late effect of virus reactivation

**Fig. 4:**
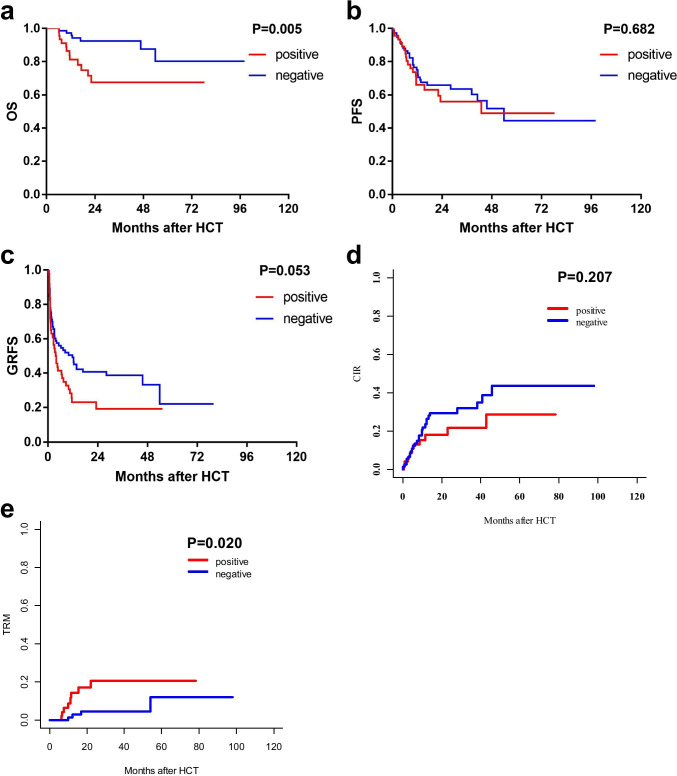
Late effect on transplant outcomes of either EBV or CMV reactivation in patients survived for more than 180 days after allo-HCT. **a** OS of patients with or without virus reactivation. **b** PFS of patients with or without virus reactivation. **c** GRFS of patients with or without virus reactivation. **d** CIR of patients with or without virus reactivation. **e** TRM of patients with or without virus reactivation

The late effect of virus reactivation was investigated in a subgroup of 120 patients who had viral reactivation within 100 days and survived for more than 180 days post-HCT. Although the incidences of relapse (2-year CIR: 21.6% ± 0.4% versus 29.5% ± 0.3%, *P* = 0.207), PFS (2-year PFS: 56.1% ± 8.1% versus 65.8% ± 5.7%, *P* = 0.682), and GRFS (2-year GRFS: 19.2% ± 6.3% versus 40.6% ± 5.8%, *P* = 0.053) were comparable, the virus reactivated group (either EBV or CMV) exhibited a significantly higher late TRM (2-year TRM: 20.1% ± 0.5% versus 4.7% ± 0.1%, *P* = 0.020), resulting in a lower OS (2-year OS: 67.6% ± 8.0% versus 92.5% ± 3.2%, *P* = 0.005) (Fig. 4).

## Discussion

Both EBV and CMV reactivations are similarly common complications as a result of HCT-induced compromised virus-specific immunity, which merits regular monitoring to avoid fatal EBV and CMV diseases. Although the growing proportion of haplo-HCT and ATG use increases the risk of virus reactivation after transplantation [25], the impact might be compensated by the advances of anti-virus agents [26, 27]. Since reports focusing on NHL patients were limited, we conducted this retrospective study to provide more data to benefit further studies in this field.

The incidence of EBV reactivation was significantly decreased in the non-LBL B-cell NHL group, which was probably attributed to the frequent application of rituximab in these patients[4, 28]. In the rituximab-treated subgroup, the only patient who developed EBV reactivation had a long interval from the last dose of rituximab to HCT (more than 1 year). In addition, we attributed the decreased incidence of EBV reactivation in T-cell LBL to fewer lines of chemotherapy (*P* = 0.001) and shorter interval from diagnosis to transplant (*P* = 0.011), since allotransplant was early recommended in most of these patients.

For EBV reactivation, HLA-mismatched donors and ATG use have been previously recognized as risk factors [25]. Recipients of HLA-mismatched donor HCT generally accept relatively stronger immunosuppressive therapy due to the HLA barrier, including ATG, which accordingly increases the risk of viral infection. In addition, we identified that previous chemotherapy of more than 6 lines and advanced disease status were independent factors for EBV reactivation. Complicated treatment courses and advanced disease status pre-HCT might indicate the aggressive nature of lymphoma, impaired hematopoietic capacity, and poorer performance. Neutrophil recovery within 30 days was an independent protective factor against EBV reactivation, which partially reflected the reservation of marrow function and fast immune reconstitution post-HCT [29, 30].

We did not find any independent risk factor for CMV reactivation in the whole cohort, which probably resulted from the homogenous transplant protocol in our center. Nevertheless, in T-cell LBL patients, the IPI and chronic GVHD were independently associated with CMV reactivation. A higher IPI score indicated a high tumor burden and worsened immune function that potentially facilitated CMV reactivation post-HCT. Meanwhile, an increased risk of chronic GVHD accompanied by CMV reactivation might result from the excessive activation of donor immune cells by CMV reactivation. Another evidence of such excessive activation was a decreased risk of CIR but an increased risk of grade 3–4 acute GVHD in CMV-positive patients in our cohort.

In accordance with our results, previous studies also demonstrated the protective effect of CMV reactivation against relapse or progression after allo-HCT in AML patients [17, 31, 32] as well as in NHL patients [33]. This protective effect might be mediated by CMV-driven expansion of donor-derived memory-like NKG2C + and NKG2D + natural killer cells, NKp46 cells, CD8 + T cells, and γ/δ T cells to intensify the graft-versus-lymphoma (GVL) effect [34–37]. However, Green et al. [32] and Mariotti et al. [38] failed to prove the protective effect of CMV reactivation after allo-HCT in a subgroup of NHL patients, so as Sawayama et al. [39] reported in 468 patients with T-cell leukemia/lymphoma. However, in all three of the abovementioned studies, CMV reactivation was monitored by pp65 antigenemia which was less sensitive than the Q-PCR method [40, 41], and a majority of recipients underwent reduced-intensity conditioning (RIC), which had a higher risk of relapse than MAC [42]. It was speculated that in patients who received RIC, host-derived memory T cells can persist for up to 6 months and contribute to immunity against CMV, preventing early expansion of donor T cells and NK cells [42, 43]. Moreover, the risk of grade III–IV acute GVHD was increased accompanied by CMV reactivation (*P* = 0.022). In our study, acute GVHD might be triggered by alloreactivity caused by the expansion of donor T cells against CMV reactivation, which led to worse TRM and GRFS.

Since the late effect of virus reactivation on transplant outcomes has been reported previously [25, 31], it was also explored in our cohort and found a poorer outcome for surviving recipients who had either EBV or CMV reactivation within 100 days post-HCT. Therefore, more effective measures should be further taken to control overt virus replication without compromising the virus-induced effect of anti-lymphoma, probably of EBV/CMV-specific cytotoxic T cells [44–46] and novel antiviral drugs [47–49].

In conclusion, we described the features of EBV and CMV reactivation after allo-HCT in patients with NHL as well as their impact on transplant outcomes. These findings of this study were restricted by several limitations, including the inherited drawbacks of a single-center retrospective study, limited sample size, mostly high-risk diseases in the cohort, and relatively homogenous transplant protocol, etc. Large-scale multicenter prospective studies are needed to validate our findings, and further research is needed to improve the treatment outcomes concerning EBV and CMV reactivation post-HCT.

## Supplementary Information

Below is the link to the electronic supplementary material.

Supplementary file1 (PDF 118 kb)

Supplementary file2 (PDF 137 kb)

Supplementary file3 (PDF 124 kb)

## Data Availability

The datasets generated during and/or analyzed during the current study are available from the corresponding author on reasonable request.
