# Supplementary material for: Epstein–Barr virus and cytomegalovirus reactivation after allogeneic hematopoietic cell transplantation in patients with non–Hodgkin lymphoma: the prevalence and impacts on outcomes: EBV and CMV reactivation post allo-HCT in NHL
Source: Ann Hematol. 2021 Sep 4;100(11):2773–85. doi: 10.1007/s00277-021-04642-5 (PMC8510926; doi:10.1007/s00277-021-04642-5)
Supplement: Supplementary file 1 — Supplementary file1 (PDF 118 kb) [file 277_2021_4642_MOESM1_ESM.pdf]

Title: Epstein–Barr virus and Cytomegalovirus reactivation after allogeneic hematopoietic cell transplantation in patients with non-Hodgkin lymphoma: the prevalence and impacts on outcomes

Journal name: Annals of Hematology

Authors: Yiyang Ding1,2,3#, Yuhua Ru1,2,3#, Tiemei Song1,2,3#, Xiang Zhang1,2,3, Jinjin Zhu1,2,3, Caixia Li1,2,3, Zhengming Jin1,2,3, Haiwen Huang1,2,3, Yuqing Tu1,2,3, Mimi Xu1,2,3, Yang Xu1,2,3, Jia Chen1,2,3*, Depei Wu1,2,3*

1. National Clinical Research Center for Hematologic Diseases, Jiangsu Institute of Hematology, The First Affiliated Hospital of Soochow University, Suzhou, China.

2. Institute of Blood and Marrow Transplantation, Collaborative Innovation Center of Hematology, Soochow University, Suzhou, China.

3.Key Laboratory of Stem Cells and Biomedical Materials of Jiangsu Province and Chinese Ministry of Science and Technology, Suzhou, China.

#These authors are co-first authors

*Corresponding authors: Jia Chen, Depei Wu

E-mail: chenjia@suda.edu.cn and wudepei@suda.edu.cn

Table S1. Variables influencing EBV and CMV reactivation in Univariate Analysis

	EBV			CMV		
	**HR**	**95%CI**	**P**	**HR**	**95%CI**	**P**
**Sex**	0.871	0.427-1.778	0.704	0.900	0.642-1.263	0.543
Male						
Female						
**Age**	0.254	0.078-0.830	0.230	0.839	0.569-1.235	0.370
<40						
≥ 40						
**Autologous HCT before allo-HCT**	2.829	0.997-8.031	0.051	2.248	0.798-6.334	0.125
NO						
YES						
**CAR-T cell therapy before allo-HCT**	0.559	0.076-4.084	0.566	0.421	0.058-3.066	0.393
NO						
YES						
**Disease status**	2.096	1.071-4.101	**0.031**	1.184	0.868-1.614	0.287
CR						
Advanced status						
**Donors type**	2.531	1.149-5.574	**0.021**	1.644	0.836-3.235	0.150
HLA-matched donors						
HLA-mismatched donors						
**Type of graft**			0.997			0.497
BM						
PB	1.111	0.327-3.772	0.866	0.874	0.247-3.098	0.853
BM+PB	1.045	0.300-3.638	0.945	1.400	0.421-4.648	0.583
dUCB			0.973	2.451	0.255-23.584	0.438

**IPI stratification**			0.255			0.371
Low risk						
Low-intermediate risk	1.587	0.599-4.210	0.353	1.632	0.615-4.330	0.325
High-intermediate risk	1.260	0.400-3.971	0.693	2.445	0.872-6.861	0.089
High risk	5.098	0.984-26.410	0.052	1.986	0.232-17.012	0.531
**NCCN-IPI stratification**			0.136			0.792
Low risk						
Low-intermediate risk	2.541	0.774-8.343	0.124	1.389	0.538-3.590	0.497
High-intermediate risk	1.076	0.217-5.334	0.928	1.279	0.390-4.190	0.685
**Ann Arbor**			0.915			0.979
I						
II	1.783	0.161-19.692	0.637	0.000		0.969
III	1.088	0.113-10.630	0.942	1.631	0.182-14.597	0.662
IV	1.057	0.144-7.763	0.957	1.465	0.200-10.698	0.707
**Time from diagnosis to HCT**	1.267	0.651-2.465	0.487	1.758	0.926-3.336	0.084
＜8m						
≥ 8m						
**Chemotherapy lines**	1.512	1.058-2.161	**0.023**	1.376	0.994-1.905	0.054
＜6						
≥ 6						
**ATG use**	4.290	1.513-12.161	**0.006**	1.548	0.757-3.166	0.232
NO						
YES						
**TBI use**	0.966	0.473-1.972	0.923	1.343	0.708-2.548	0.366
NO						
YES						
**Rituximab**	0.073	0.010-0.531	**0.010**	1.381	0.712-2.677	0.339
NO						
YES						
**Prophylactic therapy**			0.586			0.998
Ganciclovir						
Foscarnet	0.900	0.431-1.878	0.778	0.984	0.484-2.000	0.965
Acyclovir	0.568	0.198-1.662	0.302	0.978	0.416-2.301	0.959
**Neutrophil recovery within 30 days**	0.295	0.071-1.236	0.095	0.469	0.113-1.948	0.297
NO						
YES						
**Platelet recovery within 60 days**	0.626	0.293-1.341	0.229	0.922	0.439-1.939	0.831
NO						
YES						
**acute GVHD**						
None	0.882	0.453-1.715	0.711	1.463	0.782-2.740	0.234
acute GVHD						0.234
None,grade I	0.900	0.453-1.788	0.764	1.799	0.956-3.312	0.069

Grade II-IV						
**chronic GVHD**						
None	0.503	0.219-1.153	0.104	1.175	0.606-2.278	0.634
chronic GVHD						
None,limited	0.962	0.599-1.545	0.973	1.401	0.620-3.167	0.418
Extensive						

Abbreviations: NHL: non-Hodgkin lymphoma; EBV: Epstein–Barr virus; CMV: Human cytomegalovirus; CR: complete remission; BM: bone marrow; PB:peripheral blood; dUCB: double umbilical cord blood graft; IPI: the International Prognostic Index; ATG: antithymocyte globulin; TBI: total body irradiation; GVHD: graft-versus-host disease.
