# Supplementary material for: Epstein–Barr virus and cytomegalovirus reactivation after allogeneic hematopoietic cell transplantation in patients with non–Hodgkin lymphoma: the prevalence and impacts on outcomes: EBV and CMV reactivation post allo-HCT in NHL
Source: Ann Hematol. 2021 Sep 4;100(11):2773–85. doi: 10.1007/s00277-021-04642-5 (PMC8510926; doi:10.1007/s00277-021-04642-5)
Supplement: Supplementary file 2 — Supplementary file2 (PDF 137 kb) [file 277_2021_4642_MOESM2_ESM.pdf]

Title: Epstein－Barr virus and Cytomegalovirus reactivation after allogeneic hematopoietic cell transplantation in patients with non-Hodgkin lymphoma: the prevalence and impacts on outcomes

Journal name: Annals of Hematology

Authors: Yiyang Ding1,2,3#, Yuhua Ru1,2,3#, Tiemei Song1,2,3#, Xiang Zhang1,2,3, Jinjin Zhu1,2,3, Caixia Li1,2,3, Zhengming Jin1,2,3, Haiwen Huang1,2,3, Yuqing Tu1,2,3, Mimi Xu1,2,3, Yang Xu1,2,3, Jia Chen1,2,3*, Depei Wu1,2,3*

1. National Clinical Research Center for Hematologic Diseases, Jiangsu Institute of Hematology, The First Affiliated Hospital of Soochow University, Suzhou, China.

2. Institute of Blood and Marrow Transplantation, Collaborative Innovation Center of Hematology, Soochow University, Suzhou, China.

3.Key Laboratory of Stem Cells and Biomedical Materials of Jiangsu Province and Chinese Ministry of Science and Technology, Suzhou, China.

#These authors are co-first authors

*Corresponding authors: Jia Chen, Depei Wu

E-mail: chenjia@suda.edu.cn and wudepei@suda.edu.cn

Table S2. Variables influencing EBV and CMV reactivation of subgroup in Univariate and Multivariate Analysis

	**EBV**															
	**T-cell lymphoblastic lymphoma**				**Non lymphoblastic T-cell lymphoma**				**B-cell lymphoblastic lymphoma**				**Non lymphoblastic B-cell lymphoma***			
	**Univariate Analysis**		**Multivariate Analysis**		**Univariate Analysis**		**Multivariate Analysis**		**Univariate Analysis**		**Multivariate Analysis**		**Univariate Analysis**		**Multivariate Analysis**	
	**HR (95%CI)**	**P**	**HR (95%CI)**	**P**	**HR (95%CI)**	**P**	**HR (95%CI)**	**P**	**HR (95%CI)**	**P**	**HR (95%CI)**	**P**	**HR (95%CI)**	**P**	**HR (95%CI)**	**P**
**Sex:** Female vs male	0.532 (0.148-1.910)	0.333			1.271 (0.446-3.628)	0.654			0.684 (0.080-5.867)	0.729			-	NA		
**Age:** ≥40 vs ＜40	0.041 (0.000-53.365)	0.383			0.558 (0.125-2.497)	0.446			0.966 (0.113-8.283)	0.975			-	NA		
**Autologous HCT before allo-HCT:** yes vs no	19.908 (2.071-191.407)	**0.010**	3.913 (0.311-49.307)	0.291	1.174 (0.262-5.250)	0.834			6.150 (0.639-59.167)	0.116			-	NA		
**CAR-T cell therapy before allo-HCT:** yes vs	-	NA			-	NA			3.023 (0.350-26.118)	0.315			-	NA		

	**CMV**															
	**T-cell lymphoblastic lymphoma**				**Non lymphoblastic T-cell lymphoma**				**B-cell lymphoblastic lymphoma**				**Non lymphoblastic B-cell lymphoma**			
	**Univariate Analysis**		**Multivariate Analysis**		**Univariate Analysis**		**Multivariate Analysis**		**Univariate Analysis**		**Multivariate Analysis**		**Univariate Analysis**		**Multivariate Analysis**	
	**HR (95%CI)**	**P**	**HR (95%CI)**	**P**	**HR (95%CI)**	**P**	**HR (95%CI)**	**P**	**HR (95%CI)**	**P**	**HR (95%CI)**	**P**	**HR (95%CI)**	**P**	**HR (95%CI)**	**P**
**Sex:** Female vs male	0.905 (0.278-2.942)	0.868			1.100 (0.550-2.201)	0.787			0.677 (0.235-1.954)	0.471			0.805 (0.419-1.549)	0.517		
**Age:** ≥40 vs ＜40	0.042 (0.000-81.861)	0.411			1.047 (0.470-2.332)	0.910			0.185 (0.004-8.983)	0.394			0.857 (0.486-1.510)	0.594		
**Autologous HCT before allo-HCT:** yes vs no	-	NA			1.984 (0.399-9.863)	0.403			-	NA			11.463 (2.060-63.778)	**0.005**	6.271 (1.075-36.582)	0.051
**CAR-T cell therapy before allo-HCT:** yes vs	-	NA			-	NA			0.042 (0.000-1578.658)	0.555			0.545 (0.070-4.237)	0.562		

no																								
**Disease status:** advanced status vs CR	2.937 (1.025-8.413)	**0.045**	1.411 (0.786-2.530)	0.248	1.190 (0.700-2.023)	0.521	5.648 (1.022-31.200)	**0.047**	1.766 (0.655-4.759)	0.261	-	NA	0.713 (0.196-2.591)	0.607			0.933 (0.466-1.866)	0.844	1.180 (0.557-2.500)	0.665	1.891 (0.884-4.047)	0.101		
**Donors type:**																								
HLA-mismatched donors vs HLA-matched donors	1.387 (0.465-4.140)	0.557			3.458 (0.772-15.480)	0.105	1.531 (0.179-13.118)	0.697			-	NA	1.146 (0.374-3.507)	0.811			0.887 (0.433-1.815)	0.742	1.352 (0.469-3.897)	0.577	2.039 (1.059-3.926)	**0.033**	1.641 (0.247-10.915)	0.609
**Type of graft**		0.590				0.376		0.416				NA		NA				0.240		0.635		0.131		
BM	1				1		-				-		-				1		-		1			
PB	2.586 (0.318-21.052)	0.375			0.217 (0.037-1.252)	0.087	1				-		-				0.267 (0.024-2.958)	0.282	1		0.180 (0.030-1.087)	0.062		
BM+PB	1.738 (0.209-14.455)	0.609			0.279 (0.056-1.390)	0.119	0.514 (0.103-2.558)				-		-				0.323 (0.036-2.910)	0.314	1.488 (0.288-7.682)		0.559 (0.115-2.709)	0.470		
**IPI stratification**		0.061		0.135		0.330		0.911				NA		**0.039**		**0.015**		0.392		NA		0.703		
Low risk	1		1		1		1				-		1		1		1		-		1			
Low-intermediate risk	1.649 (0.332-8.186)	0.540	1.639 (0.323-8.324)	0.551	4.097 (0.529-31.757)	0.177	0.690 (0.115-4.138)	0.685			-		2.969 (0.347-25.418)	0.321	1.755 (0.201-15.346)	0.611	0.287 (0.058-1.427)	0.127	-		0.275 (0.032-2.323)	0.236		
High-intermediate risk	3.954 (0.765-20.437)	0.101	3.799 (0.725-19.912)	0.114	1.742 (0.109-27.879)	0.695	0.942 (0.085-10.422)	0.961			-		8.772 (1.056-72.906)	**0.044**	10.911 (1.298-91.719)	**0.028**	1.046 (0.174-6.274)	0.961	-		0.338 (0.039-2.955)	0.327		
High risk	19.702 (1.608-241.401)	**0.020**	16.088 (1.107-233.701)	**0.042**	10.639 (0.633-178.731)	0.100	-				-		26.842 (1.600-450.256)	**0.022**	7.371 (0.419-129.785)	0.172	-	NA	-		-	NA		
**NCCN-IPI stratification**		0.139				0.599		0.929				NA		0.394				0.060		NA		0.497		
Low risk	1				1		1				-		1				1		-		1			
Low-intermediate risk	4.243 (0.548-32.	0.167			2.534 (0.329-19	0.372	1.522 (0.177-1	0.702			-		4.163 (0.537-3	0.172			0.163 (0.036-0	0.018	-		0.391 (0.036-2.	0.266		

	880)				.516)		3.048)						2.251)		.731)				495)			
High-intermediate risk	11.230 (1.015-124.285)	0.049			1.435 (0.090-22.975)	0.798	-	0.988			-		3.751 (0.235-59.989)	0.350	0.381 (0.040-3.579)	0.405	-		0.273 (0.030-2.489)	0.250		
**Ann Arbor**		NA				NA		**0.046**		0.467		NA		NA		NA		NA		0.804		
I	-				-		1		1		-		-		-		-		1			
II	-				-		-		-		-		-		-		-		-			
III	-				-		4.201 (0.160-109.960)	0.389	4.607 (0.171-124.299)	0.364	-		-		-		-		0.944 (0.059-15.122)	0.967		
IV	-				-		0.173 (0.018-1.665)	0.129	0.712 (0.058-8.699)	0.790	-		-		-		-		0.580 (0.073-4.593)	0.606		
**Time from diagnosis to HCT:** ≥ 8m vs ＜8m	1.199 (0.705-2.037)	0.503			0.705 (0.244-2.035)	0.518	1.520 (0.649-3.559)	0.335			-	NA	1.346 (0.780-2.322)	0.286	0.898 (0.438-1.842)	0.769	2.393 (0.828-6.911)	0.107	1.120 (0.614-2.042)	0.712		
**Chemotherapy lines:** ≥ 6 vs ＜6	1.661 (0.981-2.812)	0.059	1.606 (0.894-2.886)	0.113	1.965 (0.548-7.051)	0.300	2.771 (0.943-8.141)	0.064	2.231 (0.712-6.989)	0.169	-	NA	1.300 (0.743-2.274)	0.357	0.907 (0.443-1.856)	0.788	2.669 (0.924-7.715)	0.070	1.212 (0.567-2.590)	0.620		
**ATG use:** yes vs no	7.241 (0.946-55.421)	0.057	4.286 (0.521-35.256)	0.176	5.504 (0.717-42.229)	0.101	0.699 (0.128-3.833)	0.680			-	NA	1.070 (0.329-3.476)	0.910	0.460 (0.109-1.937)	0.290	2.412 (0.290-20.051)	0.415	0.460 (0.109-1.937)	**0.041**	2.598 (0.322-20.927)	0.370
**TBI use:** yes vs no	1.165 (0.390-3.477)	0.784			0.628 (0.196-2.014)	0.434	1.047 (0.192-5.725)	0.958			-	NA	0.977 (0.301-3.175)	0.969	1.926 (0.481-7.711)	0.355	1.531 (0.342-6.855)	0.577	1.530 (0.458-5.112)	0.489		
**Rituximab:** yes vs no	-	NA			-	NA	0.411 (0.053-3.866)	0.468			-	NA	2.675 (0.345-220.772)	0.347	2.675 (0.325-22.031)	0.360	1.117 (0.216-5.766)	0.895	0.611 (0.165-2.264)	0.461		
**Prophylactic therapy**		0.406				0.616		0.845			-	NA		0.437		0.483		0.549		0.321		
Ganciclovir	1				1		1						1		1		1		1			
Foscarnet	0.446	0.298			1.174	0.773	1.082	0.931					0.372	0.198	1.704	0.515	0.366	0.360	3.412	0.133		

	(0.098-2.037)		(0.394-3.502)		(0.180-6.496)				(0.082-1.679)				(0.343-8.459)		(0.043-3.142)		(0.687-16.935)			
Acyclovir	1.678 (0.364-7.736)	0.507	0.405 (0.050-3.302)	0.399	0.549 (0.057-5.291)	0.604			-	NA			2.994 (0.499-17.948)	0.230	0.441 (0.051-3.787)	0.456	2.302 (0.421-12.592)	0.336		
**Neutrophil recovery:** yes vs no	0.304 (0.039-2.352)	0.254	-	NA	-	NA	-	NA	-	NA			-	NA	0.211 (0.024-1.894)	0.165	0.216 (0.027-1.759)	0.152		
**Platelet recovery:** yes vs no	0.482 (0.150-1.556)	0.223	0.406 (0.126-1.311)	0.132	0.223 (0.025-2.000)	0.180	-	NA	2.992 (0.389-23.026)	0.282			2.202 (0.271-17.910)	0.460	0.585 (0.070-4.897)	0.621	0.381 (0.121-1.195)	0.098	0.786 (0.216-2.866)	0.715
**acute GVHD**							-	NA												
None	1		1		1				1				1		1		1			
acute GVHD	0.467 (0.156-1.395)	0.503	1.150 (0.402-3.286)	0.794	1.585 (0.290-8.661)	0.595			1.554 (0.508-4.759)	0.440			1.839 (0.439-7.703)	0.405	0.887 (0.198-3.968)	0.875	2.266 (0.716-7.171)	0.164		
None,grade I	1		1		1				1				1		1		1			
Grade II-IV	0.712 (0.399-1.272)	0.251	1.780 (0.611-5.183)	0.290	1.333 (0.599-2.970)	0.481			1.319 (0.764-2.277)	0.321			1.333 (0.667-2.667)	0.416	1.176 (0.556-2.488)	0.671	1.505 (0.846-2.678)	0.164		
**chronic GVHD**							-	NA												
None	1		1		1				1		1		1		1		1			
chronic GVHD	0.360 (0.080-1.621)	0.183	0.482 (0.134-1.738)	0.265	1.180 (0.216-6.447)	0.848			5.280 (1.725-16.159)	**0.004**	7.814 (2.263-26.979)	**0.001**	0.876 (0.177-4.343)	0.871	0.369 (0.044-3.074)	0.357	0.325 (0.071-1.487)	0.147		
None,limited	1		1		1		-	NA	1				1		1		1			
Extensive	0.670 (0.242-1.858)	0.442	0.906 (0.252-3.261)	0.880	1.011 (0.345-2.962)	0.984			2.348 (1.339-4.119)	0.003			1.287 (0.578-2.867)	0.536	0.195 (0.002-15.683)	0.465	0.201 (0.005-8.088)	0.394		

Abbreviations: NHL: non-Hodgkin lymphoma; EBV: Epstein − Barr virus; CMV: Human cytomegalovirus; CR: complete remission; BM: bone marrow; PB:peripheral blood; dUCB: double umbilical cord blood graft; IPI: the International Prognostic Index; ATG: antithymocyte globulin; TBI: total body irradiation; GVHD: graft-versus-host disease.
