# Supplementary material for: Epstein–Barr virus and cytomegalovirus reactivation after allogeneic hematopoietic cell transplantation in patients with non–Hodgkin lymphoma: the prevalence and impacts on outcomes: EBV and CMV reactivation post allo-HCT in NHL
Source: Ann Hematol. 2021 Sep 4;100(11):2773–85. doi: 10.1007/s00277-021-04642-5 (PMC8510926; doi:10.1007/s00277-021-04642-5)
Supplement: Supplementary file 3 — Supplementary file3 (PDF 124 kb) [file 277_2021_4642_MOESM3_ESM.pdf]

Title: Epstein–Barr virus and Cytomegalovirus reactivation after allogeneic hematopoietic cell transplantation in patients with non-Hodgkin lymphoma: the prevalence and impacts on outcomes

Journal name: Annals of Hematology

Authors: Yiyang Ding1,2,3#, Yuhua Ru1,2,3#, Tiemei Song1,2,3#, Xiang Zhang1,2,3, Jinjin Zhu1,2,3, Caixia Li1,2,3, Zhengming Jin1,2,3, Haiwen Huang1,2,3, Yuqing Tu1,2,3, Mimi Xu1,2,3, Yang Xu1,2,3, Jia Chen1,2,3*, Depei Wu1,2,3*

1. National Clinical Research Center for Hematologic Diseases, Jiangsu Institute of Hematology, The First Affiliated Hospital of Soochow University, Suzhou, China.

2. Institute of Blood and Marrow Transplantation, Collaborative Innovation Center of Hematology, Soochow University, Suzhou, China.

3.Key Laboratory of Stem Cells and Biomedical Materials of Jiangsu Province and Chinese Ministry of Science and Technology, Suzhou, China.

#These authors are co-first authors

*Corresponding authors: Jia Chen, Depei Wu

E-mail: chenjia@suda.edu.cn and wudepei@suda.edu.cn

Table S3.Variables influencing Survival Outcomes in Univariate Analysis

	OS			PFS			CIR			TRM			GRFS		
	**HR**	**95% CI**	**P**	**HR**	**95% CI**	**P**	**HR**	**95% CI**	**P**	**HR**	**95% CI**	**P**	**HR**	**95% CI**	**P**
EBV: positive vs negative	1.958	0.997-3.846	0.051	1.559	0.907-2.679	0.108	1.551	0.772-3.114	0.217	1.392	0.590-3.282	0.450	1.708	1.018-2.866	**0.043**
CMV: positive vs negative	1.861	0.978-3.543	0.059	0.861	0.478-1.551	0.619	0.263	0.081-0.855	**0.026**	2.205	1.038-4.683	**0.040**	1.935	1.166-3.208	**0.011**
Sex: female vs male	0.740	0.399-1.372	0.339	0.859	0.674-1.094	0.217	0.874	0.633-1.207	0.413	0.857	0.594-1.237	0.410	1.185	0.809-1.735	0.385
Age: ＜40 vs ≥40	1.591	0.886-2.858	0.120	1.01	0.783-1.302	0.936	0.817	0.558-1.198	0.301	1.261	0.890-1.789	0.192	0.771	0.501-1.186	0.236
Autologous HCT before allo-HCT: yes vs no	1.654	0.595-4.602	0.335	1.323	0.575-3.044	0.510	0.877	0.212-3.627	0.856	2.233	0.786-6.341	0.132	1.118	0.520-2.407	0.775
CAR-T cell therapy before allo-HCT: yes vs no	1.238	0.299-5.119	0.768	1.225	0.385-3.895	0.732	0.724	0.099-5.273	0.750	1.821	0.435-7.630	0.412	1.063	0.434-2.607	0.893
Disease status: advanced status vs CR	2.315	1.31-4.092	**0.004**	1.279	1.025-1.596	**0.029**	1.1	0.818-1.481	0.528	1.564	1.106-2.212	**0.011**	1.034	0.862-1.242	0.716
Donors type: HLA-mismatched donors vs HLA-matched donors	0.926	0.529-1.620	0.787	0.963	0.771-1.204	0.743	0.906	0.670-1.225	0.521	1.07	0.767-1.483	0.690	1.06	0.881-1.275	0.536

**Type of graft**			1.000			0.982			0.939			0.998			0.753
BM	1			1			1			1			1		
PB	1.018	0.349-2.970	0.974	0.868	0.401-1.882	0.721	0.808	0.301-2.166	0.672	0.979	0.281-3.410	0.973	0.843	0.436-1.630	0.612
BM+PB	1.018	0.354-2.924	0.975	0.853	0.398-1.827	0.683	0.733	0.276-1.946	0.534	1.054	0.310-3.583	0.933	0.979	0.514-1.863	0.948
dUCB	0.000	0.000	0.977	0	0.000	0.967	0	0.000	0.975	0	0.000	0.980	0.433	0.055-3.426	0.427
**IPI stratification**			0.656			0.492			0.147			0.172			**0.020**
Low risk	1			1			1			1			1		
Low-intermediate risk	1.170	0.529-2.586	0.689	1.605	0.850-3.032	0.145	1.906	0.838-4.332	0.124	1.192	0.433-3.282	0.734	1.656	0.993-2.763	0.053
High-intermediate risk	1.570	0.658-3.748	0.309	1.458	0.706-3.011	0.308	0.799	0.268-2.384	0.687	2.298	0.819-6.453	0.114	1.446	0.807-2.593	0.215
High risk	1.941	0.411-9.159	0.402	1.994	0.562-7.079	0.286	1.152	0.141-9.385	0.895	3.184	0.617-16.438	0.167	4.664	1.716-12.674	**0.003**
**NCCN-IPI stratification**			0.407			0.116			0.091			0.435			0.228
Low risk	1			1			1			1			1		
Low-intermediate risk	1.819	0.716-4.623	0.208	1.826	0.906-3.680	0.092	1.667	0.701-3.964	0.247	2.123	0.641-7.035	0.218	1.581	0.912-2.738	0.102
High-intermediate risk	1.404	0.445-4.425	0.562	1.12	0.455-2.756	0.806	0.509	0.127-2.041	0.341	2.303	0.595-8.911	0.227	1.28	0.638-2.566	0.487
**Ann Arbor**			0.389			0.536			0.542			0.280			0.639
I	1			1			1			1			1		
II	0.194	0.020-1.872	0.156	0.303	0.055-1.658	0.169	0.586	0.037-9.382	0.706	0.22	0.023-2.116	0.190	0.616	0.154-2.464	0.493
III	0.475	0.133-1.998	0.310	0.634	0.190-2.113	0.458	2.076	0.249-17.322	0.500	0.229	0.038-1.373	0.107	0.598	0.184-1.946	0.393
IV	0.399	0.123-1.294	0.126	0.556	0.203-1.533	0.257	1.219	0.167-8.905	0.845	0.343	0.104-1.130	0.079	0.873	0.321-2.374	0.791
Time from diagnosis to HCT: ≥8m vs ＜8m	1.56	0.889-2.737	0.121	1.089	0.874-1.356	0.446	0.903	0.672-1.214	0.500	1.436	1.013-2.037	**0.042**	1.029	0.859-1.233	0.753
Chemotherapy lines: ≥ 6 vs＜6	1.569	0.890-2.765	0.119	1.434	0.920-2.237	0.112	1.428	0.789-2.584	0.239	1.385	0.708-2.709	0.342	1.14	0.950-1.367	0.158
Rituximab: yes vs no	1.371	0.756-2.484	0.299	1.179	0.726-1.914	0.506	0.73	0.351-1.515	0.398	1.969	1.000-3.875	0.050	1.183	0.791-1.771	0.413
**Prophylactic therapy**			0.532			0.159			0.374			0.323			0.753
Ganciclovir	1			1			1			1			1		
Foscarnet	1.393	0.747-2.597	0.297	1.611	0.989-2.625	0.056	1.553	0.819-2.947	0.178	1.676	0.787-3.566	0.181	1.168	0.778-1.754	0.453
Acyclovir	1.346	0.635-2.857	0.438	1.293	0.704-2.374	0.407	1.036	0.440-2.440	0.935	1.689	0.703-4.056	0.241	1.051	0.635-1.739	0.848

Neutrophil recovery: yes vs no	0.136	0.053-0.349	**<0.001**	0.194	0.083-0.451	**<0.001**	0.288	0.088-0.939	**0.039**	0.247	0.075-0.815	**0.022**	0.273	0.126-0.590	**0.001**
Platelet recovery: yes vs no	0.238	0.133-0.426	**<0.001**	0.312	0.193-0.504	**<0.001**	0.506	0.252-1.016	0.056	0.207	0.104-0.412	**<0.001**	0.65	0.429-0.983	**0.041**
ATG use: yes vs no	0.777	0.441-1.370	0.383	0.769	0.491 -1.206	0.253	0.696	0.384-1.263	0.233	0.868	0.436-1.728	0.686	0.805	0.553-1.170	0.255
TBI use: yes vs no	1.63	0.929-2.859	0.088	1.259	0.798-1.988	0.323	1.46	0.803-2.654	0.215	1.028	0.503-2.100	0.939	0.997	0.681-1.460	0.987
acute GVHD	0.817	0.467-1.430	0.480	0.985	0.791-1.226	0.891	0.989	0.738-1.325	0.941	0.982	0.705-1.369	0.913			
none,grade I vs grade II	1.061	0.602-1.871	0.837	0.968	0.771-1.214	0.777	0.841	0.613-1.154	0.283	1.145	0.821-1.598	0.425			
chronic GVHD	0.233	0.099-0.552	**0.001**	0.895	0.684-1.121	0.291	1.124	0.830-1.522	0.450	0.589	0.366-0.949	**0.030**			
none,limited vs extensive	0.393	0.141-1.102	0.076	0.616	0.306-1.238	0.174	0.741	0.312-1.756	0.495	0.464	0.140-1.534	0.208			

Abbreviations: NHL: non-Hodgkin lymphoma; EBV: Epstein－Barr virus; CMV: Human cytomegalovirus; CR: complete remission; BM: bone marrow; PB:peripheral blood; dUCB: double umbilical cord blood graft; IPI: the International Prognostic Index; ATG: antithymocyte globulin; TBI: total body irradiation; GVHD: graft-versus-host disease.
